# Randomised controlled comparison of the Health Survey Short Form (SF-12) and the Graded Chronic Pain Scale (GCPS) in telephone interviews versus self-administered questionnaires. Are the results equivalent?

**DOI:** 10.1186/1471-2288-7-50

**Published:** 2007-11-22

**Authors:** Margitta Lungenhausen, Stefan Lange, Christoph Maier, Claudia Schaub, Hans J Trampisch, Heinz G Endres

**Affiliations:** 1 Department of Pain Management, BG-Kliniken Bergmannsheil, Ruhr University Bochum, D-44789 Bochum, Germany; 2 Institute for Quality and Efficiency in Health Care, Dillenburger Str. 27, D-51105 Cologne, Germany; 3 Department of Medical Informatics, Statistics and Epidemiology, Ruhr University Bochum, D-44801 Bochum, Germany

## Abstract

**Background:**

The most commonly used survey methods are self-administered questionnaires, telephone interviews, and a mixture of both. But until now evidence out of randomised controlled trials as to whether patient responses differ depending on the survey mode is lacking. Therefore this study assessed whether patient responses to surveys depend on the mode of survey administration. The comparison was between mailed, self-administered questionnaires and telephone interviews.

**Methods:**

A four-armed, randomised controlled two-period change-over design. Each patient responded to the same survey twice, once in written form and once by telephone interview, separated by at least a fortnight. The study was conducted in 2003/2004 in Germany. 1087 patients taking part in the German Acupuncture Trials (GERAC cohort study), who agreed to participate in a survey after completing acupuncture treatment from an acupuncture-certified family physician for headache, were randomised. Of these, 823 (664 women) from the ages of 18 to 83 (mean 51.7) completed both parts of the study. The main outcome measure was the comparison of the scores on the 12-Item Short-Form Health Survey (SF-12) and the Graded Chronic Pain Scale (GCPS) questionnaire for the two survey modes.

**Results:**

Computer-aided telephone interviews (CATI) resulted in significantly fewer missing data (0.5%) than did mailed questionnaires (2.8%; p < 0.001). The analysis of equivalence revealed a difference between the survey modes only for the SF-12 mental scales. On average, reported mental status score was 3.5 score points (2.9 to 4.0) lower on the self-administered questionnaire compared to the telephone interview. The order of administration affected results. Patients who responded to the telephone interview first reported better mental health in the subsequent paper questionnaire (mean difference 2.8 score points) compared to those who responded to the paper questionnaire first (mean difference 4.1 score points).

**Conclusion:**

Despite the comparatively high cost of telephone interviews, they offer clear advantages over mailed self-administered questionnaires as regards completeness of data. Only items concerning mental status were dependent on the survey mode and sequence of administration. Items on physical status were not affected. Normative data for standardized telephone questionnaires could contribute to a better comparability with the results of the corresponding standardized paper questionnaires.

## Background

The survey methods most commonly used in clinical trials are self-administered questionnaires (SAQ), telephone interviews (TI), and a mixture of both ("mixed-mode method") that consists of mailing the self-administered questionnaires and following up by telephoning non-respondents. The highest response rates are generally achieved either with telephone interviews or with the mixed-mode method, both of which tend to minimise complete drop-outs and missing values for individual items [1-5]. It is known that patients who do not respond to mailed questionnaires, on average report greater dissatisfaction with treatment when contacted by telephone than do those who mail back their questionnaires [5,6]. A recently published study comparing the telephone-administration mode of the SF-36 with the self-administered mode concluded that the telephone-administration mode is equivalent to and as valid as the self-administered mode[7].

By contrast, in designing the present study we hypothesized that patients respond differently to questions about psychological states than to those about physical symptoms. The latter will probably be answered more honestly, because physical problems are more socially accepted[8,9]. For our study we therefore chose to compare patient responses to the 12-Item Short-Form Health Survey (SF-12) [10] and the Graded Chronic Pain Scale (GCPS) questionnaire[11] – two widely used survey instruments that collect data on both mental and physical aspects of pain disorders – in the telephone interview mode and the self-administration mode. A test-retest design was selected to examine whether the order of administration and/or the preliminary information of half the respondents had any effect on patient response behaviour in the comparison of SAQ and TI.

The test-retest design was chosen in order to test memory effects. It is conceivable, for example, that subjects would be better able to memorize their answers in one of the survey modes, thus resulting in greater similarity in responses between the first and second measurements. The point in time at which subjects are informed that they would be asked to answer a second questionnaire could affect results in a similar way. For example, subjects concerned about social acceptance and wishing to give very precise answers might, if told ahead of time that they would be asked to respond to more than one questionnaire, use memory aids such as making notes before the questionnaires were administered.

## Methods

### Design

**Figure 1:**
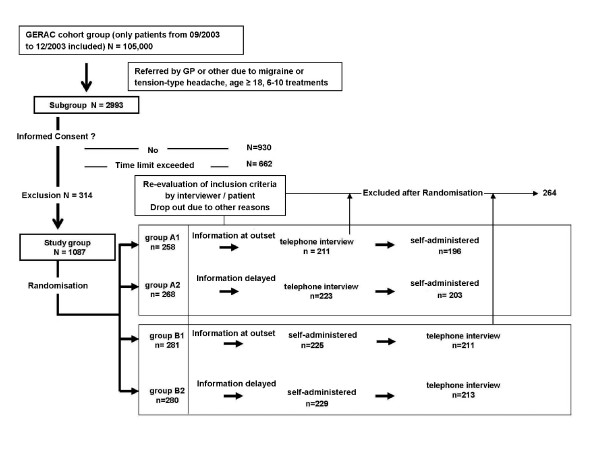
Study design.

A four-armed, randomised controlled two-period change-over design (Figure 1) was used. Each patient responded to the same survey twice, once in written form (SAQ) and once by telephone interview (TI), separated by at least a fortnight. This obligatory minimum interval between administration of the two survey modes made bias due to recall of previous answers very unlikely. Patients were first randomly assigned to one of the two main groups A (TI first) or B (SAQ first), and then to one of two subgroups within each main group (A1 and A2 or B1 and B2). Groups A1 and B1 were informed ahead of time that a second survey would be administered, while groups A2 and B2 were not. All patients who participated in both TI and SAQ were included in the evaluation (Figure 1).

The study was approved by the local ethic committee of the Ruhr-University Bochum.

### Participants

The study was conducted in 2003/2004. Participants were drawn from our GERAC acupuncture trial and consisted of a random sample of cohort patients who had received acupuncture treatment from an acupuncture-certified physician, in many cases their family physician [12,13]. The sample was selected based on the patient case report forms submitted by the treating physician, documenting the patient's demographic data, acupuncture course, and acupuncture indication. Primary eligibility criteria were age 18 years or older, acupuncture treatment for migraine and/or tension-type-headaches, and at least six acupuncture treatments received (most had had ten). Patients who were eligible and willing to participate were randomly assigned to the four groups (Figure 1). After randomisation patients were contacted by telephone to inform them about the study procedures, evaluate the correctness of acupuncture indication, assess whether patients had sufficient cognitive and linguistic capacities to participate, and schedule the TI or mailing of the SAQ.

### Instruments

The GCPS is a standard self-assessment instrument used in medical pain research and quality management that offers a means of hierarchically classifying chronic pain severity independent of the pain syndrome[11]. In this study the scores "pain intensity" and "pain-related disability" were analysed. The scores range from 0 to 100, with 100 being maximum pain intensity or disability. The SF-12 measures patients' physical and mental state of health on two separate scales[10]. SF-12 scores range from 0 to 100, with 100 being complete absence of impairment.

### Data collection

All study procedures were guided by an Oracle^®^-based software developed especially for the GERAC cohort study. The system was used to manage interview appointments, conduct interviews using electronic case report forms (eCRFs), and transfer responses from paper questionnaires to the Oracle^® ^data base. Data completeness was verified by a software routine that displayed warning notices for missing data.

Steps were taken to ensure that the interval between administration of the two questionnaires was at least two weeks. For groups A1 and A2 (TI first), the paper questionnaire was mailed 10 working days after the administration of the telephone interview (allowing several days for mail delivery), with instructions to complete the questionnaire immediately upon receipt. In groups B1 and B2 (SAQ first), participants received the paper questionnaire at an agreed on date within a six-week period. The second interview (TI) was conducted at least two weeks after the paper questionnaire had been returned by mail.

All telephone interviews including the first contact followed standardised interview guidelines, and used the same wording as the paper questionnaire items wherever possible. In the case of the SF-12, the interview version was used. Interviews were conducted on weekdays between 9:00 am and 7:00 pm. Interviewers received several days of pre-study training on study design and interview techniques and were supervised by psychologists at all times. The interviews were conducted by 20 students of Ruhr-University Bochum.

### Statistics

An ANOVA design was used to test for equivalence and differences between the two survey modes (TI or SAQ) in two different steps. Equivalence was examined using the confidence-interval inclusion rule [14]. Equivalence was assumed if the 90% confidence interval for the mean difference between the factor steps to be tested (the survey modes) was found to lie within a 25% standard deviation, with interval limits based on statistical values of a standardised collective. For the SF-12, those values lie within a limit of ± 2.5 score points for both scales. Since no standardised data are available for the GCPS, we accessed a database of the German Society for the Study of Pain (DGSS) that stores data for at present 1465 patients suffering from migraine or chronic tension-type-headaches. The mean score for pain intensity in this collective was 73.70 (SD 17.42). For upper and lower limits of ± 0.25 standard deviation, the interval limit for assuming equivalence was rounded and defined as ± 5 score points. The differences for the conditions "survey-mode sequence" in the second step and "point in time when patients were informed of second survey" in the third step were tested using the F-test. Missing data were analysed by means of chi-squared tests. All analyses were performed using SPPS 12 for Windows.

## Results

### Participants

Of the 2993 primary eligible patients, 930 (31.1%) did not respond to the invitation or declined to participate without giving reasons, while 662 (22.1%) responded, but too late. Patients who declined to participate cited various personal reasons (242), health reasons (14), time reasons (31), or communication problems (hearing loss, bad knowledge of German (15). 314 (10.5%) respondents who agreed to participate had to be excluded because they were treated for indications different from those stated on the CRF. Initially 1087 (36.3%) could be randomised (Figure 1), of whom 823 (76.2%) completed the whole study. 125 (11.5%) patients dropped out because they preferred not to continue, 122 (11.2%) were excluded because it was determined in the course of the interview that they had not in fact received acupuncture treatment for headache, and another 17 (1.6%) were excluded due to partial deafness or insufficient knowledge of German. The number of patients excluded any time after randomisation was almost the same in all four groups. [A1: 62 (18.2%), A2: 65 (16.9%), B1: 70 (19.9%), B2: 67 (18.2%), p = 0.92], and was independent of when patients were informed about the second survey (p = 0.87). The final sample consisted of 664 female and 159 male participants (Table 1), aged 18 to 83, with an average age of 51.7.

**Table 1:** Gender and indication of sample

	Mean Age (SD); Median	Migraine (%)	Tension-type headache (%)	Mixed diagnosis: Migraine/tension-type headache (%)	Total (%)
Male	55.8 (13.5) 58.4	44 (5.4)	110 (13.4)	5 (0.6)	159 (19.3)
Female	50,7 (14.2), 50.8	258 (31.3)	384 (46.6)	22 (2.7)	664 (80.7)
Total	51.7 (14.2); 51.6	302 (36.7)	494 (60.0)	27 (3.3)	823 (100.0)

### Missing data

Only completely answered questionnaires were included in the final analysis. In the case of the SF-12, missing data occurred in a total of 120 data sets, resulting in exclusion from analysis [SAQ: 113 (13.7%), TI: 7 (0.9%)]. In the case of the GCPS, 25 data sets had to be excluded [SAQ: 19 (2.3%), TI: 6 (0.7%)]. Analysis of the incomplete questionnaires showed that there were more missing values in the SAQs than in the TIs [SAQ total: 414 (2.8%) vs. TI total: 67 (0.5%); p < 0.001], and a higher rate of missing items for the SF-12 than for the GCPS [SF-12 total 347 (3.5%) vs. GCPS total 134 (2.7%); p = 0.01]. The frequency of missing data was also higher for the SF-12 mental scales than for the physical scales [SF-12 mental: 211 (4.3%), SF-12 physical: 176 (3.6%); p = 0.07]. Again, missing rates were lower in the TIs [SF-12 mental: 43 (0.9%), SF-12 physical: 37 (0.7%); p = 0.5].

### Testing for equivalence

The analysis of equivalence revealed a difference between survey modes for the SF-12 mental scales (Table 2). Patients reported poorer mental status on the SAQ than in the TI (mean difference 3.5; 90% confidence interval (CI) 2.9 to 4.0). By contrast the 90% confidence interval for the mean difference of the SF-12 physical scale was within the limits of ± 2.5 score points (mean difference 1.8; 90% CI 1.3 to 2.3). The mean differences for the two GCPS subscales also lie within the GCPS limits of ± 5 score points (mean difference GCPS pain intensity 0.3; 90% CI -0.7 to 1.2; mean difference GCPS pain-related disability -3.2; 90% CI -4.4 to 2.0) (Table 3).

**Table 2:** Condition-based mean values (M) and standard deviation (SD) for SF-12: mental (SF-12m) and physical (SF-12p) scales

		**Telephone interview**		**Self-administered questionnaire**		
			
**Timing of information about second survey**	**Survey-mode sequence**	**SF-12p **M (SD)	**SF-12m **M (SD)	**SF-12p **M (SD)	**SF-12m **M (SD)	**N**
Delayed	Self-administered questionnaire first	45.22 (10.0)	49.10 (10.6)	43.21 (10.0)	44.69 (11.3)	183
	Telephone interview first	44.83 (11.0)	49.79 (9.8)	42.67 (10.6)	46.93 (10.5)	178
	Total	45.03 (10.5)	49.44 (10.2)	42.94 (10.3)	45.79 (11.0)	361
At outset	Self-administered questionnaire first	43.91 (10.1)	48.19 (11.0)	42.32 (9.6)	44.35 (11.3)	183
	Telephone interview first	43.68 (10.2)	49.80 (10.1)	42.35 (9.8)	47.01 (10.5)	164
	Total	43.80 (10.2)	48.95 (10.6)	42.33 (9.7)	45.61 (11.0)	347
Total	Self-administered questionnaire first	44.57 (10.1)	48.65 (10.8)	42.77 (9.8)	44.52 (11.3)	366
	Telephone interview first	44.28 (10.6)	49.80 (9.9)	42.51 (10.2)	46.97 (10.5)	342
	Total	44.43 (10.3)	49.20 (10.4)	42.64 (10.0)	45.70 (11.0)	708

**Table 3:** Condition-based mean values (M) and standard deviation (SD) for GCPS: pain intensity (PI) and pain-related disability (PD) scales

		**Telephone interview**		**Self-administered questionnaire**		
			
**Timing of information about second survey**	**Survey-mode sequence**	**PI **M (SD)	**PD **M (SD)	**PI **M (SD)	**PD **M (SD)	**N**
Delayed	Self-administered questionnaire first	55.56 (19.3)	31.20 (25.7)	55.56 (19.8)	34.53 (26.7)	205
	Telephone interview first	52.32 (19.9)	26.75 (25.4)	52.68 (19.7)	30.96 (24.7)	194
	Total	53.99 (19.7)	29.03 (25.7)	54.16 (19.8)	32.79 (25.7)	399
At outset	Self-administered questionnaire first	56.39 (17.8)	31.06 (24.9)	55.34 (20.4)	34.55 (25.7)	203
	Telephone interview first	56.00 (20.9)	30.75 (26.2)	55.68 (20.2)	32.37 (24.3)	190
	Total	56.20 (19.3)	30.91 (25.5)	55.50 (20.3)	33.51 (25.0)	393
Total	Self-administered questionnaire first	55.97 (18.6)	31.13 (25.3)	55.45 (20.1)	34.54 (26.2)	408
	Telephone interview first	54.14 (20.5)	28.71 (25.8)	54.17 (20.0)	31.65 (24.5)	384
	Total	55.08 (19.5)	29.96 (25.6)	54.83 (20.0)	33.15 (25.4)	792

### Testing for difference by survey mode sequence

**Figure 2:**
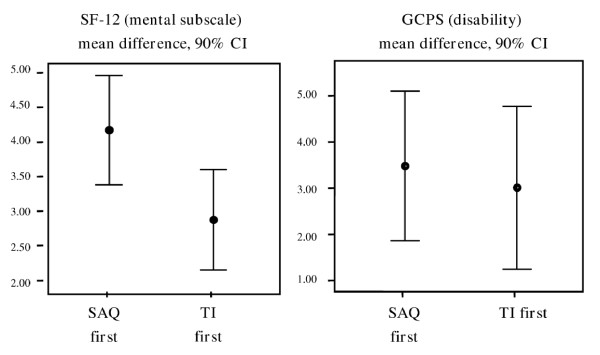
Mean differences between the two survey modes SAQ vs. TI, by survey-mode sequence.

Survey mode sequence affected response behaviour in the second survey for the SF-12 mental scales only. Patients who responded to the TI first reported better mental health in the following SAQ on average. The same effect was not observed in the reverse sequence. The mean difference between the two survey modes was greater for the condition "SAQ first" than for the condition "TI first" [SF-12 mental scales: mean difference: 4.1 (SAQ first) vs. 2.8 (TI first); 90% CI 0.2 to 2.4, p < 0.05]. There were no other statistically relevant differences, either as regards the SF12 physical scales or the GCPS subscales [SF-12 physical scales: mean difference 1.8 (SAQ first) vs. 1.8 (TI first); 90% CI -0.9 to 1.0, p > 0.05; GCPS pain intensity: -0.5 (SAQ first) vs. 0.03 (TI first); 90% CI -2.4 to 1.3, p > 0.05; GCPS pain-related disability: 3.4 (SAQ first) vs. 2.9 (TI first); 90% CI -1.9 to 2.9, p > 0,05)] (Figure 2).

The point in time when patients were informed about the second survey had no effect on the level of agreement between the two survey modes: SF-12 physical scales: mean difference -2.1 (delayed information) vs. -1.4 (information at outset); 90% CI -0.6 to 1.6, p > 0.05); SF-12 mental scales: 3.6 vs. 3.4; 90% CI -0.8 to 1.4, p > 0.05); GCPS pain intensity: 0.2 vs. -0.7; 90% CI -1.0 to 2.7, p > 0.05; GCPS pain-related disability: 3.8 vs. 2.6; 90% CI -1.2 to 3.5, p > 0.05).

## Discussion

The purpose of this study was to assess the agreement between results of telephone interviews and self-administered mailed questionnaires for the SF-12 and the GCPS, two important instruments in clinical research and practice, with the help of a two-period change-over design. To our knowledge no such data have ever been obtained for the GCPS. The study should provide insight as to whether patients' response behaviour is influenced by motivational aspects, information that would be useful for planning clinical and epidemiological trials.

The results of equivalency testing show that the response behaviour of chronic pain patients is subject to different motivational mechanisms, depending on whether the questions concern mental or physical health. Patients gave a more positive estimation of their mental health in telephone interviews than in the self-administered questionnaires. The same was not true for the SF-12 physical scales or the GCPS subscales. The most likely explanation is that the taboo that society still places on mental disability seems to cause patients speaking with another person in a telephone interview to minimise mental problems that accompany physical illness. This tendency is less likely to affect responses to the more anonymous self-administered questionnaires.

Another result of this study is that computer-assisted telephone interviews have clear advantages over mailed self-administered questionnaires when it comes to completeness of data. In addition to this previously recognized advantage of telephone interviews, however, we found that the number of missing responses was closely related to question content. There tended to be more missing responses for items concerning mental status than for those relating to physical condition. These findings support the hypothesis that patients suffering from chronic pain often view their illness as purely physical and therefore shy away from answering questions about their mental state. In the telephone interview, on the other hand, the trained interviewer is able to obtain substantially more complete responses.

A third result of this study is that, in contrast to previous findings [2,15,16], the level of agreement between SF-12 scores in TI and SAQ mode for the mental health subscale was dependent on the survey mode sequence. The differences were more marked if the patient had first given a more positive assessment of mental status in the telephone interview. These results can be explained with the help of findings from the psychology of memory (e.g. [17]): a more positive assessment of mental health status in the telephone interview is associated in the respondent's memory with more positive emotions, which facilitate retrospective recall of memory content when the patient subsequently fills out the paper questionnaire. A more negative assessment of physical health would have the reverse effect: negative emotions block recall of memory content, making it more likely that the respondent will describe his or her current physical state in the subsequent survey. The point in time when patients were informed about the second survey had no effect on response behaviour. Evidently the announcement to participants that they will be asked to complete a second survey is understood as information at a formal level only. Cognitive and emotional processing related to estimating one's own state of health is not likely to be influenced by when this information is received.

The strengths of our study are the comparison of GCPS values in the two survey modes, and the new control variable "Point in time when patients were informed of second survey." To our knowledge neither of these has done before.

One limitation of our study is that we cannot rule out a real remission of symptoms in the interval between the administration of the two surveys, which was at least 14 days, and therefore cannot rule out the possibility that response behaviours were influenced by real symptom improvements.

Another limitation is the non-response rate of close to 25%, as the response behaviour of this group could well differ from that of the rest of the study population. However, a systematic bias resulting from a high non-response rate is more likely to occur when the purpose of the survey is to measure treatment success, which was not the case in the present study. A more generous time frame for the return of questionnaires might increase the response rate. In this study, 662 patients did not return the questionnaire until after the four-week period allotted. The average time taken by those patients to return the questionnaire was seven weeks.

There are also some practical disadvantages to using the CATI-System for data collection: depending on the population size and the technical equipment available, this mode is more time consuming and more costly than mailing out paper questionnaires. Administration of surveys via the Internet might be a cost-effective alternative. A factor to be considered, however, is that proportionately fewer people have access to this means of communication than to the telephone.

Chronic pain research and therapy has traditionally been an interdisciplinary undertaking. Besides quality of life questionnaires, other important data gathering instruments are questionnaires that measure fear or depression. Using instruments such as the CES-D in telephone interviews[18] could, by analogy to results obtained for the mental health subscale of the SF-12 in our study, lead to a systematic underestimation of depression.

Since telephone interviews offer significant advantages over self-administered questionnaires, further mode-comparing studies in this area, particularly with chronic pain patients, are clearly needed.

## Conclusion

The most commonly used method of collecting data from patients is still the self-administration of a paper questionnaire. But telephone interviews are being more widely used because of the markedly better data quality obtained by this means. Until now RCT evidence as to whether patient responses differ depending on the survey mode has been lacking. We strongly recommend that mixing of questionnaire modes should be avoided when gathering data with respect to mental health criteria. When a homogeneous questionnaire mode is used, the reliability of responses should theoretically not be affected, since deviations will always be in the same direction. However, outcomes may not be directly comparable to those of other studies if the data were gathered by means of a different mode. Normative data for standardized telephone questionnaires could contribute to a better comparability with the results of the corresponding standardized paper questionnaires.

## Competing interests

The author(s) declare that they have no competing interests.

## Authors' contributions

All authors commented on the draft and the interpretation of the findings, read and approved the final manuscript. ML was responsible for the telephone interviews and supervision, progress of the study, analysis, interpretation and reporting of the data, and wrote the original manuscript; SL was responsible for conception and design, analysis and interpretation of data and presentation of results; CM was responsible for conception and design, interpretation of the data and expertise in pain treatment; CS was responsible for interpretation of the data, telephone interviews and expertise in evaluation of questionnaires; HJT was responsible for conception and design, statistical analysis plan, and statistical expertise; HGE was principal investigator, responsible for the study protocol, conception and design, progress of the study, analysis and interpretation of the data.

## Pre-publication history

The pre-publication history for this paper can be accessed here:


